# Demonstration of brain region-specific neuronal vulnerability in human iPSC-based model of familial Parkinson’s disease

**DOI:** 10.1093/hmg/ddaa039

**Published:** 2020-03-11

**Authors:** Razvan-Marius Brazdis, Julian E Alecu, Daniel Marsch, Annika Dahms, Katrin Simmnacher, Sandra Lörentz, Anna Brendler, Yanni Schneider, Franz Marxreiter, Laurent Roybon, Beate Winner, Wei Xiang, Iryna Prots

**Affiliations:** 1 Department of Stem Cell Biology, Friedrich-Alexander-Universität Erlangen-Nürnberg, Erlangen 91054, Germany; 2 Department of Psychiatry and Psychotherapy, University Hospital Erlangen, Friedrich-Alexander-Universität Erlangen-Nürnberg, Erlangen 91054, Germany; 3 Institute of Biochemistry (Emil-Fischer-Center), Friedrich-Alexander-Universität Erlangen-Nürnberg, Erlangen 91054, Germany; 4 Department of Molecular Neurology, University Hospital Erlangen, Friedrich-Alexander-Universität Erlangen-Nürnberg, Erlangen 91054, Germany; 5 Stem Cell Laboratory for CNS Disease Modeling, Department of Experimental Medical Science, Lund University, Lund 22184, Sweden

## Abstract

Parkinson’s disease (PD) is a neurodegenerative disorder characterized by protein inclusions mostly composed of aggregated forms of α-synuclein (α-Syn) and by the progressive degeneration of midbrain dopaminergic neurons (mDANs), resulting in motor symptoms. While other brain regions also undergo pathologic changes in PD, the relevance of α-Syn aggregation for the preferential loss of mDANs in PD pathology is not completely understood yet. To elucidate the mechanisms of the brain region-specific neuronal vulnerability in PD, we modeled human PD using human-induced pluripotent stem cells (iPSCs) from familial PD cases with a duplication (Dupl) of the α-Syn gene (SNCA) locus. Human iPSCs from PD Dupl patients and a control individual were differentiated into mDANs and cortical projection neurons (CPNs). SNCA dosage increase did not influence the differentiation efficiency of mDANs and CPNs. However, elevated α-Syn pathology, as revealed by enhanced α-Syn insolubility and phosphorylation, was determined in PD-derived mDANs compared with PD CPNs. PD-derived mDANs exhibited higher levels of reactive oxygen species and protein nitration levels compared with CPNs, which might underlie elevated α-Syn pathology observed in mDANs. Finally, increased neuronal death was observed in PD-derived mDANs compared to PD CPNs and to control mDANs and CPNs. Our results reveal, for the first time, a higher α-Syn pathology, oxidative stress level, and neuronal death rate in human PD mDANs compared with PD CPNs from the same patient. The finding implies the contribution of pathogenic α-Syn, probably induced by oxidative stress, to selective vulnerability of substantia nigra dopaminergic neurons in human PD.

## Introduction

Parkinson’s disease (PD) is the most common neurodegenerative movement disorder affecting around 1% of the population aged over 65 years (1). Neuropathologically, PD is characterized by the progressive degeneration of dopaminergic neurons in the substantia nigra (SN) of the midbrain (2). The resulting progressive loss of nigrostriatal dopamine signaling is the main cause of the cardinal motor symptoms of PD, such as bradykinesia, rigidity and resting tremor (3). Besides, emotional and cognitive disturbances, contributing to the non-motor symptoms of PD, are based on the dysregulation of cortical circuits, which are partly dependent on the midbrain dopamine system (4–7).

Another neuropathological hallmark of PD is the deposition of intracellular inclusions, mainly consisting of pathologically aggregated α-synuclein (α-Syn) in neuronal cell bodies and neurites, called Lewy bodies (LBs) and Lewy neurites (LNs), respectively (8). α-Syn is a small, physiologically soluble protein, predominantly located at the presynaptic site, which aggregates in brains of PD patients. The aggregation of α-Syn proceeds through monomers and aggregation intermediates (such as oligomers and protofibrils) to insoluble fibrils, which finally deposit along with other components, forming LBs and LNs (9). Among aggregated α-Syn species, oligomers were suggested to be the most toxic species (10,11), causing neurite pathology and dysregulation of axonal transport, whose disruption is strongly connected to neuronal cell death (12,13). In addition to α-Syn deposition in diseased brains, α-Syn gene (SNCA) mutations and multiplications of the SNCA locus cause early onset genetic forms of PD (14), further strengthening the pathological importance of α-Syn for PD. Thus, PD patients carrying the SNCA locus duplication (Dupl) are characterized by the presence of a widespread Lewy pathology from the brainstem to the neocortex and neuronal loss in the SN (15).

Analysis of human post-mortem brain tissues suggests a predictable PD-specific spreading pattern of α-Syn pathology during disease progression, initiating in the brainstem and progressing toward the midbrain and, later, to the neocortex (16). Despite of the widespread of Lewy pathology in various neuronal types in PD brains, preferential degeneration of midbrain dopaminergic neurons (mDANs) in PD suggests their particular vulnerability. Although the underlying mechanisms are yet unclear, several interconnected pathways may contribute to the selective vulnerability of mDANs: (1) mDANs produce the neurotransmitter dopamine that is highly prone to self-oxidation, leading to the generation of reactive oxygen species (ROS), implying a high basal oxidative stress level in mDANs (17); (2) oxidative stress is known as a risk factor of α-Syn oligomerization (18), suggesting that the intracellular milieu of mDANs facilitates the aggregation of α-Syn; and (3) since α-Syn is involved in dopamine synthesis, release, and reuptake (19), α-Syn aggregation may impact dopamine homeostasis, by e.g. stimulating dopamine reuptake, thereby exacerbating oxidative stress and leading to cellular damage (20).

In this study, we aimed to address the vulnerability of mDANs and the correlation to α-Syn pathology in human PD by employing human-induced pluripotent stem cell (iPSC)-derived neurons. To study the selective vulnerability of mDANs in PD, we compared mDANs and cortical projection neurons (CPNs) differentiated from iPSCs of PD patients with SNCA Dupl and of a healthy donor. We observed an elevated α-Syn aggregation propensity in PD mDANs compared with both, control mDANs and CPNs, but even more importantly compared with PD CPNs from the same patient. Coincidently, mDANs showed higher levels of ROS and protein nitration, when compared with CPNs, indicating an increased oxidative stress level in mDANs. Consequently, PD Dupl mDANs were characterized by a higher basal neuronal death, resembling a selective loss of mDANs in PD brain. Our results demonstrate, for the first time, a stronger vulnerability and a more pronounced α-Syn pathology of mDANs compared with CPNs from the same PD patients with SNCA Dupl. These results support the role of α-Syn aggregation, probably potentiated by SNCA dosage increase and oxidative stress, in promoting the selective vulnerability of dopaminergic neurons in the SN in human PD.

## Results

### Cortical and midbrain neuronal differentiation is not altered by the SNCA dosage increase

To address the selective vulnerability of mDANs and detect whether they exhibit a PD-related pathology, we generated mDANs and CPNs from iPSCs of a heathy donor (Ctrl) and a PD patient carrying a Dupl of the SNCA locus (PD Dupl). To avoid genetic variabilities (21), we used identical iPSC lines of the PD Dupl patient and of the healthy donor for the differentiation of both neuronal subtypes. We previously demonstrated that CPNs differentiated from the same iPSC line of the PD Dupl patient exhibit impaired axonal transport, accompanied by an increased formation of α-Syn aggregation intermediates as compared with Ctrl CPNs (12).

**Figure 1:**
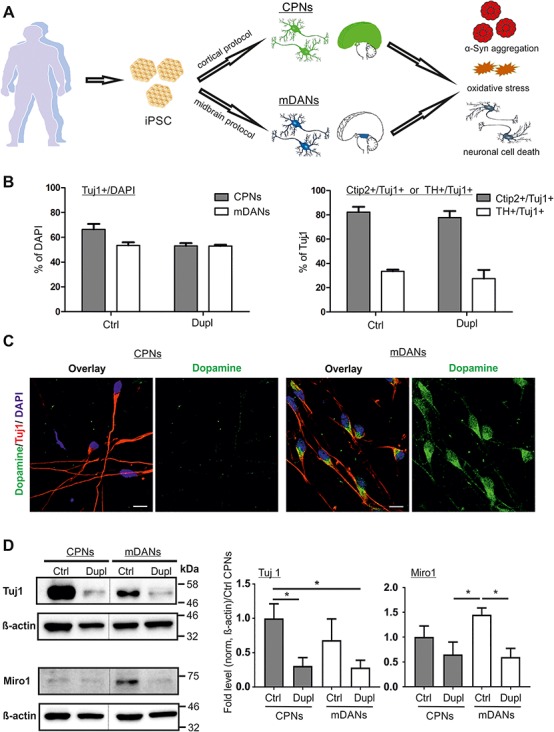
α-Syn gene (SNCA) locus duplication (Dupl) does not alter neuronal differentiation efficiency. (**A**) Schematic summary of the study. CPNs and mDANs were differentiated from human iPSCs of a control individual (Ctrl) and PD patients carrying SNCA Dupl (Dupl) using cortical and small-molecule-based midbrain protocols, respectively. α-Syn aggregation was analyzed biochemically. Oxidative stress was evaluated by the level of ROS and protein nitration. Neuronal cell death was determined by ICC analysis of cleaved Caspase-3 (C-Casp3). (**B**) Neuronal differentiation was equally effective in Ctrl and Dupl as assessed by ICC of neuronal marker β3-tubulin (Tuj1), cortical projection marker Ctip2 and midbrain dopaminergic marker TH (representative images used for the quantification are shown in Fig. 4A). Amounts of neurons were determined as the percentage (%) of Tuj1-positive cells over DAPI-positive cells, whereas cortical and midbrain neurons were evaluated as the proportion (%) of Ctip2- or TH-positive neurons over total Tuj1-positive neurons in three independent differentiation rounds. Values are shown as mean ± SD. Two-tailed Student’s *t*-test was used. (**C**) ICC staining of dopamine in iPSC neuronal cultures revealed positive dopamine signals in Tuj1-positive neurons, differentiated from Ctrl iPSCs by the midbrain protocol, while dopamine was barely detectable in Ctrl CPNs, confirming the neurotransmitter-specific phenotype of mDANs. Scale bars 10 μm. (**D**) Tuj1 and Miro1 protein expression was assessed in Ctrl and PD Dupl CPNs and mDANs by WB. Quantification was conducted by the normalization of Tuj1 or Miro1 signals to β-actin levels followed by setting Ctrl CPN levels to 1. Reduced Tuj1 and Miro1 expression was determined in both PD Dupl CPNs and mDANs compared with Ctrl neurons. ^*^P ≤ 0.05; values are shown as mean ± SD (three independent experiments). One-way ANOVA with multiple comparisons test was used. Blots for Tuj1 and Miro1 within one black frame are derived from the same membrane. β-Actin was used as a loading control and was probed on the same membrane as the respective target protein. Lanes from different parts of the same membrane are separated by black dashed lines.

IPSCs from a healthy control and the PD Dupl patient were differentiated into mDANs and CPNs (Fig. 1A). Due to the involvement of fibroblast growth factor (FGF) signaling pathways in the forebrain and midbrain patterning (22), CPNs were differentiated by using FGF2 as a modulator of proliferation and differentiation of the neural precursor cells (NPCs) (23), according to a previously published protocol (24). mDANs were generated by a combination of FGF8, known to promote dopaminergic differentiation (25), and small molecules to inhibit signaling pathways of transforming growth factor-β and bone morphogenetic protein (26).

Neuronal differentiation was assessed by immunocytochemistry (ICC), based on the expression of β3-tubulin (Tuj1), a pan neuronal marker, and was similarly effective in Ctrl and PD Dupl cultures, resulting in 53–66% of Tuj1-positive neurons generated by both cortical and midbrain protocols (Fig. 1B, left panel; representative ICC images used for the quantification are shown in Fig. 4A). Human iPSC differentiation into CPNs and mDANs was evaluated using antibodies (Abs) against the CPN marker—COUP-TF-interacting protein 2 (Ctip2) and the mDAN marker—tyrosine hydroxylase (TH). Cortical and midbrain differentiation efficiencies were comparable between Ctrl and PD Dupl iPSC lines, reaching about 80% Ctip2-positive CPNs and about 30% TH-positive mDANs, respectively (Fig. 1B, right panel; representative ICC images used for the quantification are shown in Fig. 4A). These data indicate that SNCA dosage increase has no major impact on either neuronal or neurotransmitter-specific differentiation efficiency. Importantly, dopamine staining revealed dopamine-positive cells among Tuj1-positive neurons, differentiated by the midbrain protocol, while Tuj1-positive neurons, developed by the cortical protocol, showed only the marginal signals of dopamine, confirming a neurotransmitter-specific phenotype of iPSC-derived neurons, in particular mDANs (Fig. 1C).

Although Ctrl and PD Dupl iPSC lines differentiated equally well into CPNs and mDANs, the total β3-tubulin level was reduced in PD Dupl-derived neurons, either in CPNs or mDANs, when compared with respective neurons derived from Ctrl iPSCs (Fig. 1D, Tuj1 panel). Given the comparable differentiation efficiency of neurons in both Ctrl and PD Dupl cultures (as analyzed by the percentages of Tuj1-positive cells, Fig. 1B), these results suggest reduced β3-tubulin level per individual PD Dupl neurons compared with Ctrl neurons. Consistently, the levels of mitochondrial Rho GTPase 1 (Miro1), required for a proper development, morphology and intracellular distribution of mitochondria and associated with PD pathology (27), were decreased in PD Dupl-derived neurons (Fig. 1D, Miro1 panel). Taken together, in accordance with neuronal impairments in PD Dupl CPNs described previously in our study (12), mDANs from the same PD Dupl iPSC also showed neuronal deficits.

### Increased high molecular weight α-Syn oligomers in PD Dupl mDANs

**Figure 2:**
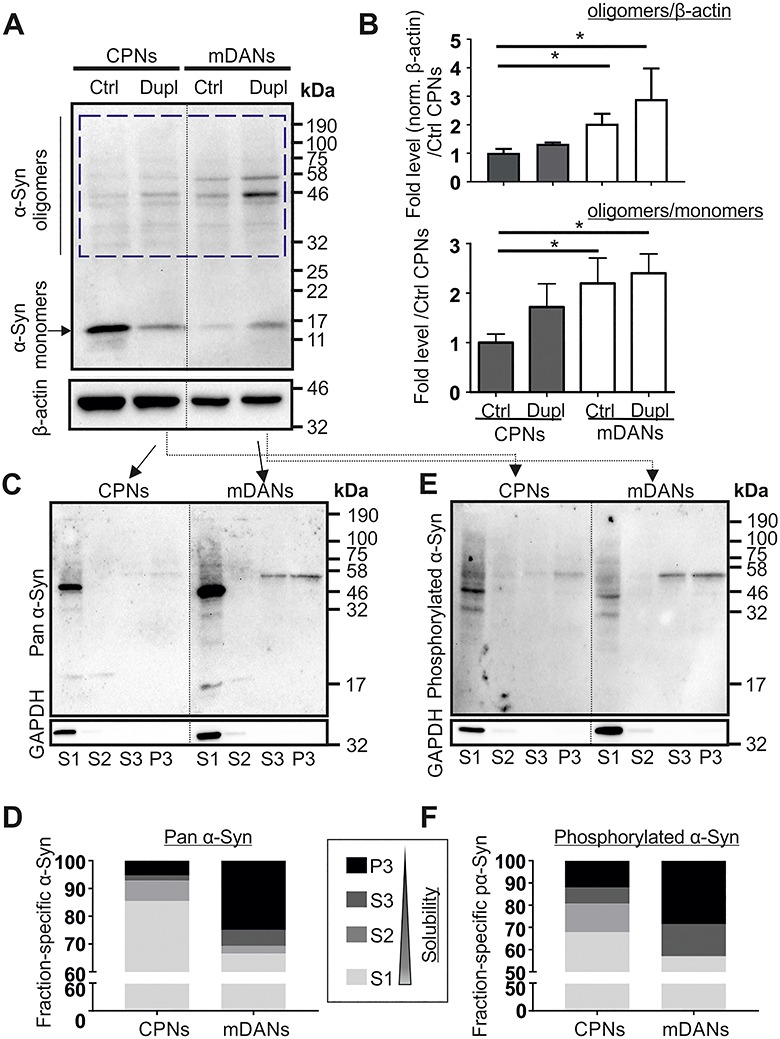
Higher α-Syn aggregation levels in mDANs compared with CPNs in PD Dupl case. (**A**) High molecular weight SDS-stable α-Syn oligomers were determined by WB using a pan α-Syn antibody (Syn1). β-Actin was used as a loading control and was probed on the same membrane. (**B**) α-Syn oligomer level in each sample was quantified by measuring the signal intensity in the region ranging from 32 kDa (corresponding to the molecular weight of α-Syn dimers) to 190 kDa (blue dashed box in A). Quantifications were performed by the normalization of signals either to β-actin levels (in order to determine the levels of α-Syn oligomers) or to α-Syn monomer (to analyze relative ratios of α-Syn oligomers to monomers in each sample) followed by setting Ctrl CPN levels to 1 (upper and lower diagrams, respectively). PD Dupl mDANs exhibit the highest α-Syn oligomer levels as shown in the representative WB (A) and by quantifications (B). Numbers on the right of the WB panel represent molecular weights of a protein ladder in kDa. Values are shown as mean ± SD of three independent experiments. ^*^P ≤ 0.05 by one-way ANOVA. (C–F) The solubility of α-Syn was determined by sequential extraction of proteins, followed by WB analysis of α-Syn distribution in fractions carrying proteins with decreasing solubility (solubility S1 > S2 > S3 > P3 fractions). GAPDH, a soluble cytosolic protein, was probed on the same membranes for α-Syn to control the sequential extraction. α-Syn and phosphorylated α-Syn (S129) in different fractions were probed using (**C** and **D**) a pan α-Syn antibody and (**E** and **F**) a phosphorylated α-Syn antibody, respectively. Solubility analysis reveals a decreased α-Syn solubility and increased formation of insoluble phosphorylated α-Syn species. A prominent band between 46 and 58 kDa found in S3 and P3 fractions derived from PD Dupl mDANs represents phosphorylated oligomeric α-Syn. Quantification shown in (D) and (F) was done by calculating the proportion of α-Syn positive signals (from monomeric and oligomeric α-Syn) in each fraction. Blots for α-Syn within one black frame (in C and E) are derived from the same membrane. GAPDH was probed on the same membrane as α-Syn. Lanes from different parts of the same membrane are separated by black dashed lines.

We next investigated whether the level of α-Syn aggregation differs in CPN and mDAN populations and whether α-Syn pathology is especially evident in neurons from the PD Dupl patient. To these aims, whole cell lysates of CPNs and mDANs derived from Ctrl and PD Dupl were analyzed by denaturing sodium dodecyl sulfate-polyacrylamide gel electrophoresis (SDS-PAGE) followed by western blot (WB) for determining the detergent-stable higher molecular weight α-Syn oligomers that appear between 32 kDa (the molecular weight of dimers on SDS-PAGE) and 190 kDa (Fig. 2A, blue dashed box). To evaluate the level of SDS-stable α-Syn oligomers, α-Syn oligomer immunosignals were normalized either to β-actin for determining the fold changes of oligomers (Fig. 2B, upper diagram) or to α-Syn monomers for determining the ratio changes associated with monomer-to-oligomers transition (Fig. 2B, lower diagram). We observed that both total and proportional α-Syn SDS-stable oligomer levels are generally higher in mDANs than in CPNs (Fig. 2B). Whereas a tendency of increased α-Syn oligomers in PD Dupl mDANs compared with PD Dupl CPNs was found, a significant increase in α-Syn oligomer levels was detected in PD Dupl and Ctrl mDANs compared with Ctrl CPNs (*P* ≤ 0.05, Fig. 2B). An increase of α-Syn oligomers in mDANs compared with CPNs suggests a particular predisposition of mDANs to form neurotoxic α-Syn species.

To further verify an enhanced oligomerization propensity, we performed sequential extraction of proteins, which allows to separate insoluble α-Syn (in S3 and P3 fractions) from soluble (in S1 fraction) and less soluble or vesicle-bound α-Syn (in S2 fraction) under native conditions prior to denaturing SDS-PAGE. In order to determine differences between mDNAs and CPNs related to PD pathology, we focused on comparing α-Syn solubility in PD Dupl neurons. We detected a proportional increase of insoluble α-Syn species in PD Dupl mDANs compared with PD Dupl CPNs using a pan α-Syn antibody (Fig. 2C and D). In agreement with a reduced solubility of total α-Syn, the proportional levels of α-Syn phosphorylated at the amino acid residue S129 were also increased in insoluble fractions generated by sequential extraction of proteins (Fig. 2E and F). Phosphorylation of α-Syn at S129 is closely related to PD and was found in LBs and LNs (28). Therefore, phosphorylation of α-Syn is widely used as an indicator for α-Syn aggregation, especially for oligomerization (29). Notably, the levels of oligomeric species with a molecular weight of 46–58 kDa showed a remarkable increase in insoluble S3 and P3 fractions in PD Dupl mDANs (Fig. 2C). Interestingly, these insoluble oligomeric species were also detected in the same samples using an antibody against phosphorylated S129 α-Syn (Fig. 2E), indicating that prominent oligomeric α-Syn bands correspond to insoluble phosphorylated species.

In summary, complementary aggregation analyses revealed an increased aggregation propensity of α-Syn in PD mDANs compared with PD CPNs, characterized by a higher level of α-Syn oligomers, enhanced insolubility and a proportional increase of insoluble phosphorylated α-Syn.

### Increased levels of ROS and protein nitration in PD Dupl mDANs

**Figure 3:**
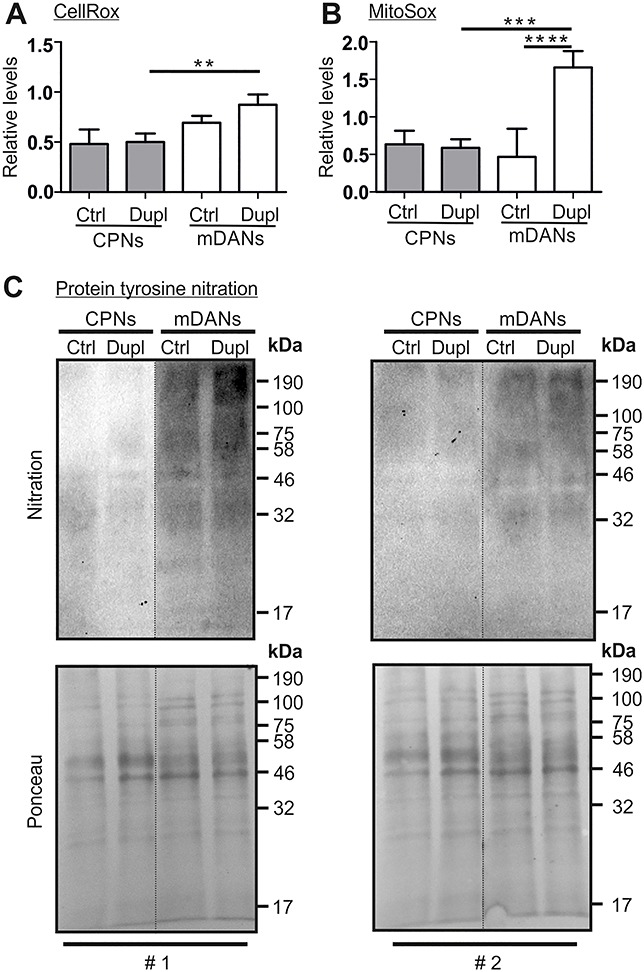
Higher oxidative stress levels in mDANs compared with CPNs. (**A**) Cytosolic ROS and (**B**) mitochondrial superoxide were measured in Ctrl and PD Dupl CPNs and mDANs by the CellRox reagent and MitoSox, respectively, using a CLARIOStar Plus plate reader. CellRox and MitoSox signals were normalized to respective DAPI signals followed by the normalization to an external CPN and mDAN control line. Significantly higher ROS levels both (A) cytosolic and (B) mitochondrial were determined in PD Dupl mDANs compared with PD Dupl CPNs. (**C**) Total protein nitration level was determined in CPNs and mDNAs generated from Ctrl and PD Dupl iPSCs by WB using an antibody against nitrotyrosine. The blots were stained by Ponceau for protein loading control prior to immunodetection. Protein nitration levels are remarkably higher in mDANs compared with CPNs. Blots with samples from two independent differentiation experiments (#1 and #2) are shown. Blots for nitration within one black frame are derived from the same membrane. Ponceau staining was performed on the same membrane prior to the nitration detection. Lanes from different parts of the same membrane are separated by black dashed lines.

The comparison between mDANs and CPNs demonstrates a higher α-Syn aggregation tendency in PD Dupl mDANs with a particularly strong effect when compared with PD Dupl CPNs. As mDANs are assumed to have high oxidative stress levels due to active dopamine metabolism, we next asked whether the degree of oxidative stress in different neuronal subtypes correlates with distinct α-Syn aggregation patterns. Oxidative stress is characterized by the overproduction of ROS and reactive nitrogen species (RNS). Therefore, we first measured the levels of ROS in CPNs and mDANs, respectively, derived from Ctrl and PD Dupl. We applied CellRox and MitoSox fluorescent reagents to assess cytosolic ROS and mitochondrial superoxide, respectively. CellRox and MitoSox fluorescence intensities were measured in living neurons using a multimode microplate reader in order to assure fast and simultaneous detection in multiple neuronal lines. Moreover, to exclude any possible effects of components in different neuronal differentiation media used for mDANs and CPNs on ROS levels, all subtypes of neurons were cultured in the same minimal essential neuronal media for the last 48 h prior to fluorescence detection. Significantly higher cytosolic ROS and mitochondrial superoxide levels were determined in PD Dupl mDANs compared with PD Dupl CPNs (*P* < 0.01 for CellRox and *P* < 0.001 for MitoSox, Fig. 3A and B, respectively). Of note, MitoSox, but not CellRox, levels were significantly higher in PD Dupl mDANs when compared with Ctrl mDANs, suggesting a more prominent mitochondrial pathology in PD Dupl mDANs (*P* < 0.0001, Fig. 3B). Altogether, these results indicate neuronal subtype-specific, higher ROS levels in PD Dupl mDANs, which are independent on the differentiation protocol.

ROS/RNS can react with susceptible amino acid residues of proteins, thereby triggering post-translational modification of proteins, such as tyrosine nitration (30). We additionally examined protein nitration levels biochemically by using an antibody against nitrotyrosine and could show that mDANs, derived either from Ctrl or from PD Dupl iPSCs, exhibit markedly higher protein nitration levels (Fig. 3C). Together with our data from ROS analyses (Fig. 3A and Fig. 3B), protein nitration results demonstrate a higher oxidative stress level in mDANs compared with CPNs, which was especially pronounced in PD Dupl mDANs.

### Increased cell death rate in mDANs compared with CPNs in PD Dupl cases

**Figure 4:**
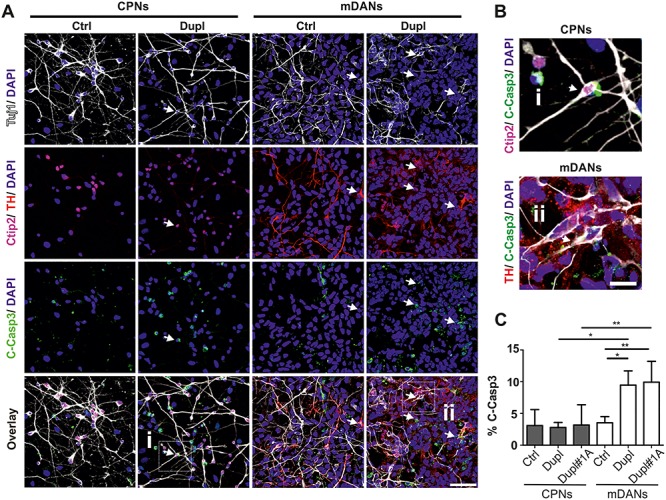
Increased apoptosis in mDANs compared with CPNs in PD Dupl cases. (**A**) iPSC-derived CPNs (Tuj1+/Ctip2+) and mDANs (Tuj1+/TH+) from the control individual (Ctrl) and PD Dupl patients (Dupl) were stained for cleaved Caspase-3 (C-Casp3) to determine neuronal death rate in respective neurons. Representative images used for neuronal subtype differentiation efficiency (Fig. 1B) and for the C-Casp3 (Fig. 4C) quantifications are shown. Arrows indicate the examples of C-Casp3+ neurons. (**B**) Enlarged views of selected cells marked by white frames in (A), representing a Tuj1+/Ctip2+/C-Casp3+ cell (i) and a Tuj1+/TH+/C-Casp3+ cells (ii). (**C**) Quantification of ICC analysis. iPSC-derived CPNs and mDANs from two PD Dupl patients (Dupl and Dupl#1A) were analyzed for neuronal cell death. Control (Ctrl) combines data from CPNs or mDANs, independently differentiated from two different iPSC clones of the same healthy individual. Significantly higher apoptosis rates were detected in mDANs from both PD Dupl patients (Dupl and Dupl#1A; % of TH+/C-Casp3+ over Tuj1+) compared with Ctrl, as well as to CPNs (% of Ctip2+/C-Casp3+ over Tuj1+) of PD Dupl cases. DAPI visualized cell nuclei. Values are shown as mean ± SD of three independent differentiations. ^*^P ≤ 0.05, ^*^^*^P ≤ 0.01 by one-way ANOVA. Scale bar 50 μm in (A) and 12.5 μm in (B).

Since PD Dupl mDANs were characterized by a particularly high levels of α-Syn pathology and oligomers compared with CPNs, we asked whether different neuronal types (mDANs versus CPNs) derived from Ctrl and PD Dupl iPSCs exhibit different basal neuronal death rate. For this, cell death was evaluated by ICC of the cleaved Caspase-3 (C-Casp3), a marker for early stages of apoptosis (31), in neurons double-positive for Tuj1 and a respective neuronal marker (Ctip2 or TH; Fig. 4A and B). IPSC-derived CPNs and mDANs from two different PD Dupl patients (Dupl and Dupl#1A) were examined for neuronal cell death. While no significant differences were found between Ctrl and PD Dupl CPNs (3.1 versus 2.9% [Dupl] and 3.2% [Dupl#1A] of C-Casp3/Ctip2-double positive neurons, respectively, *P* > 0.05), significantly elevated proportion of apoptotic PD Dupl mDANs compared with Ctrl was detected (9.5% [Dupl] and 9.9% [Dupl#1A] versus 3.5% of C-Casp3/TH-double positive neurons, respectively, *P* ≤ 0.05, Fig. 4C). Moreover, significantly higher neuronal death was determined in mDAN cultures compared with CPN cultures of both PD Dupl patients (*P* ≤ 0.05 for Dupl neurons and *P* ≤ 0.01 for Dupl#1A neurons, Fig. 4C). Altogether, these results demonstrate higher vulnerability of mDANs compared with CPNs, in particularly those derived from pathological PD Dupl iPSCs. The same holds true for α-Syn aggregation potential, which is higher in PD Dupl mDANs compared with PD Dupl CPNs and might underlie a particular vulnerability of mDANs in PD.

## Discussion

mDANs are a heavily affected neuronal population in PD. Although the selective loss of midbrain TH-positive neurons has been shown in post-mortem PD brains and in various animal PD models, preferential loss of mDANs in PD compared with other neuronal subtypes and the underlying mechanisms are less investigated, in particular in human disease models. In this study, using human iPSC-based disease modeling, we detected higher levels of α-Syn aggregation in mDANs compared with CPNs in familial PD. α-Syn pathology in PD Dupl mDANs was characterized by decreased α-Syn solubility, a proportional increase of detergent-stable oligomers and the formation of insoluble, phosphorylated oligomeric α-Syn species. Moreover, we clearly demonstrated in our model system that ROS and protein nitration levels, both indicators of oxidative stress, are higher in mDANs, particularly in PD Dupl mDANs, than in CPNs. Importantly, pronounced α-Syn pathology and high oxidative stress levels in PD Dupl mDANs were coincident with a higher neuronal death when compared with PD Dupl CPNs and Ctrl neurons. Taken together, by directly comparing human-derived neurons, we demonstrate that mDANs are particularly vulnerable in human familial form of PD with SNCA Dupl, probably due to the susceptibility of this neuronal type to SNCA dosage increase and oxidative stress, both are risk factors for α-Syn aggregation. Our results might provide an explanation of a selective vulnerability of SN neurons in human PD.

The neuronal differentiation from human iPSCs in our study yielded in total 53–66% Tuj1-positive neurons. The neuronal differentiation efficiency in our study is in accordance with previously published data using human iPSC-based disease modeling (24,26). Moreover, neuronal generation from iPSCs was comparable between Ctrl and PD Dupl iPSCs, indicating that gene dosage increase of SNCA does not impair neuronal differentiation. This finding suggests that there might be no major defects of neuronal development in PD Dupl cases. Similar to the total neuronal numbers, Ctrl and PD Dupl iPSCs were equally well differentiated to CPNs and mDANs, indicating that SNCA Dupl does not compromise neurotransmitter-specific development. In line with our results, previous studies reported the similar efficiency of cortical glutamatergic differentiation (32) and midbrain dopaminergic neuronal differentiation of iPSCs from healthy individuals (33), idiopathic (34) or genetic PD patients (26,35,36).

A widespread cortical Lewy pathology and the development of dementia are characteristic for the majority of PD patients carrying SNCA Dupl (15). Cortical Lewy pathology is known to be associated with cognitive impairment in PD Dupl patients (37). Despite the presence of Lewy pathology in the cortex of PD patients, no PD-specific neuronal loss in the cortex was found based on the investigations of post-mortem brain tissues (38). In our study, the survival rate of CPNs from healthy control and PD Dupl patients was not significantly different. Thus, although α-Syn pathology may be responsible for neuronal dysfunction in the cortical regions of PD patients resulting in dementia symptoms, our results suggest a higher resistance of CPNs to PD-driving factors such as α-Syn pathology. Due to the fact that PD is characterized by a broad Lewy pathology localization and at the same time by a neuronal loss evident only in limited brain regions, including subsets of brain nuclei and the SN, it is indeed supposed that there might be common features rendering PD pathology-related neurons more vulnerable (reviewed in 39).

In contrast to CPNs and in agreement with neuronal loss in the SN in PD, a significantly enhanced cell death rate was found in PD Dupl mDANs compared with Ctrl mDANs and PD Dupl CPNs, indicating a particular vulnerability of mDANs in human PD pathology. Thus, our results from the iPSC-based model of familial PD recapitulate a prominent and selective loss of dopaminergic neurons in the SN of PD patients. Higher death rates of mDANs from PD Dupl patients compared with Ctrl mDANs are in accordance with previous data: iPSC-based studies, comparing mDNAs generated from Ctrl and familial PD patients with either SNCA triplication or point mutations in the genes LRRK2, PARKIN or PINK1, also pointed out an increased neuronal death and elevated susceptibility of mDANs to dopamine-induced oxidative stress in PD mDNAs (40–44). Moreover, increased extracellular-signal-regulated kinase phosphorylation (26), mitochondrial dysfunctions and decreased dopaminergic differentiation were reported in mDANs from familial PD patients (45,46). Studies analyzing human post-mortem brain tissue from idiopathic PD patients (47) or from familial PD patients with SNCA Dupl (15) showed an elevated mDAN death level. All these previous findings suggest that despite widespread pathological changes of PD in different areas of the brain, the most heavily affected neurons in PD are mDANs in pars compacta of the SN.

The vulnerability of mDANs may be attributed to their intrinsic milieu, characterized by a high basal oxidative stress level and the related high α-Syn aggregation propensity. Oxidative stress is an important player of α-Syn aggregation. One underlying mechanism involves the increased formation of α-Syn oligomers under oxidative stress via α-Syn modification by ROS/RNS or oxidative stress metabolites (18,48,49). The accumulation of oxidative stress-induced modified oligomeric α-Syn species has been shown to be neurotoxic in a variety of cell models. For example, our previous study revealed that specific oxidative stress-mediated α-Syn oligomers are selectively toxic to human mDNAs (differentiated LUHMES cells) (18). All these converging findings suggest a causative link between α-Syn oligomerization and the death of mDANs in PD. An increased α-Syn expression or accumulation has been previously shown in iPSC-derived mDANs from PD patients carrying SNCA triplication (40,45,50,51) or LRRK2 mutation (41,42). Here, by comparing PD Dupl mDANs and CPNs directly, we could show that an increase of SNCA dosage preferentially promotes α-Syn aggregation in PD Dupl mDANs. The α-Syn aggregation pattern in this particular neuronal subtype is characterized by a proportional increase of SDS-stable α-Syn oligomers, a remarkable decline of α-Syn solubility and increased formation of insoluble, phosphorylated α-Syn oligomers. In contrast, neurons with a lower death rate (Ctrl CPNs, Ctrl mDANs and PD Dupl CPNs) showed lower α-Syn aggregation levels. Using various biochemical procedures, we have previously shown an increased formation of α-Syn aggregation intermediates in PD Dupl CPNs compared with Ctrl CPNs (12). Combining the results of the current study, α-Syn aggregation propensity in PD Dupl mDANs appears to be even higher than in PD Dupl CPNs.

Our analysis of oxidative stress by directly assessing ROS levels in living neurons revealed significantly higher ROS levels in PD Dupl mDANs compared with PD Dupl CPNs. Moreover, significant differences in mitochondrial superoxide levels between PD Dupl mDANs and Ctrl mDANs suggest a stronger mitochondrial pathology in PD Dupl mDANs. Evaluation of protein nitration level showed a generally higher degree in mDNAs in comparison with CPNs, regardless of their origin (Ctrl or PD Dupl), which was coincident with a more pronounced α-Syn pathology in mDANs. Our study suggests that both the distinct intracellular milieu of mDANs (oxidative stress) and abnormal α-Syn levels are important for the selective vulnerability of mDANs. One intriguing contributor to oxidative stress in mDANs could be dopamine metabolism in this specific neuronal subtype, which is supported by the clear difference in intracellular dopamine between mDANs and CPNs from the control iPSCs in our model system. Dopamine is an unstable neurotransmitter and highly prone to oxidation. During its self-oxidation, free radicals are produced, contributing to the high basal oxidative stress level. Indeed, dopamine treatment was shown to result in the darkening of midbrain spheroids containing mDANs generated from a PD patient carrying the variation p.Q811R in POLG1 gene, but not control (52). This phenotype suggests dopamine oxidation and neuromelanin deposition in mDANs and was associated with metabolic dysfunction and occurrence of high molecular weight α-Syn oligomers in PD mDANs (52). Furthermore, a progressive accumulation of oxidized dopamine was reported in iPSC-derived mDANs from sporadic and a number of different genetic PD cases, including SNCA triplication, suggesting that this phenotype might be a common pathology in both sporadic and familial forms of PD (53).

Interestingly, mitochondrial oxidative stress and oxidized dopamine were previously shown to start a pathological cascade resulting in α-Syn accumulation in PD iPSC-derived mDANs (53). Our findings of a pronounced α-Syn pathology coincident with a higher oxidative stress level in mDANs further strengthen the role of α-Syn aggregation in human PD pathology. Beside several known toxic cellular mechanisms, aggregated α-Syn species, such as oligomers, may specifically trigger mDAN death. They might mediate this effect either via modulating dopamine reuptake and homeostasis, thereby increasing oxidative stress (19) and α-Syn own aggregation. Alternatively, α-Syn oligomers may destroy the membrane integrity, resulting in either inducing mitochondrial pathology (12) or increasing abnormal Ca^2+^ currents (10) that would modulate the pacemaker firing pattern of neurons in the SN (54). Further studies using strategies to inhibit α-Syn oligomer formation (55) applied to PD mDANs could clarify this question.

In conclusion, our study shows a higher α-Syn aggregation level preferentially in mDANs, derived from a familial PD patient with SNCA locus Dupl, when compared with CPNs of the same patient and mDANs and CPNs from a control donor. We proved higher oxidative stress levels in mDANs compared with CPNs. Moreover, PD Dupl mDANs were characterized by the reduced survival rate. Thus, by directly comparing mDANs and CPNs from the identical individuals, our study suggests that α-Syn pathology, induced by an increased SNCA dosage and oxidative stress, renders vulnerability to mDANs in human PD pathology.

## Materials and methods

### Cells and cell culture

Human iPSC lines from two PD patients carrying an SNCA Dupl were kindly provided by Professor Galasko (line SDi1-R-C3 previously described (12), referred here as Dupl) and by Dr Roybon (line CSC-1A, previously described in (56), referred here as Dupl#1A). Both PD Dupl patients were female and had a disease onset at the age of 58 (Dupl) and 53 years (Dupl#1A), respectively. Both PD patients had a progressive disease course characterized by tremor, muscle cramps and dementia. The duplication of SNCA was confirmed in iPSCs from both PD patients using Multiplex Ligation-Dependent Probe Amplification analysis (P051/P052 probe mixes, MRC-Holland), which revealed SNCA dosage increase of three copies corresponding to a heterozygous duplication. IPSCs from an age- and sex-matched healthy Caucasian individual with no history of neurologic disease served as a control (lines UKERi33Q-R1–06 and UKERi33Q-R1–002). PD Dupl and Ctrl line UKERi33Q-R1–06 were used for all experiments, while neuronal death levels were additionally determined in neurons derived from PD Dupl, PD Dupl#1A and both Ctrl UKERi33Q-R1–06 and UKERi33Q-R1–002 lines. Human iPSCs were reprogrammed from fibroblasts by retroviral transduction with SOX2, KLF4, c-MYC and OCT3/4, differentiated through NPCs into CPNs or mDANs by either applying a FGF2-based cortical protocol (24) or a FGF8- and small molecule-based midbrain protocol, respectively, as previously described (26,57).

All experiments with human iPSC-derived cells were carried out in accordance with the local Institutional Review Board approval (No. 259_17B, University Hospital Erlangen, Friedrich-Alexander-Universität Erlangen-Nürnberg, Erlangen, Germany) and national and European Union directives. Written informed consents were received from the participants prior to inclusion into the study at the movement disorder clinic at the Department of Molecular Neurology, University Hospital Erlangen (Erlangen, Germany) and at the Department of Neurosciences, UCSD (San Diego, CA, USA). The iPSC line CSC-1A was generated from fibroblasts obtained with informed consent and after ethical committee approval at the Parkinson institute in Milan, Italy. The permit for reprogramming of CSC-1A was delivered by the Swedish work environment authority and registered under the number 20200-3211.

### Immunocytochemistry

Cells were fixed with 4% paraformaldehyde (PFA) for 30 min at 37°C, permeabilized by treatment with a mixture of ethanol: acetic acid (2:1) for 5 min at −20°C (for staining of all target proteins except dopamine, where this step was omitted), washed three times with phosphate-buffered saline (PBS, Invitrogen) and subsequently permeabilized/blocked by incubating in ICC blocking solution (0.3% Triton X100, 3% donkey serum [both from Sigma-Aldrich] in PBS) for 45 min at room temperature (RT). Primary Abs: anti-ß3-tubulin (Tuj1; 1:500; BioLegend), anti-TH (C-20, 1:500; Santa Cruz Biotechnology), anti-Ctip2 (25B6; 1:500; Abcam), anti-C-Casp3 (Asp175, 1:500; Cell Signaling) and anti-dopamine (ab6427; 1:1000; Abcam) were incubated in the ICC blocking solution overnight at 4°C. Fluorescently labeled secondary Abs (1:500, all from Thermo Fisher Scientific) were incubated in the ICC blocking solution 1 h at RT. Cell nuclei were stained with 1 μg/ml 4′,6-diamidino-2-phenylindole (DAPI, Sigma-Aldrich). Coverslips were mounted on glass microscope slides (Thermo Fisher Scientific) in Aqua Polymount (Polysciences). Images were acquired using a ZEISS LSM 780 confocal laser scanning microscope and Zen Software. Images were quantified using the cell counter plugin and the ImageJ software1.51j (NIH). In total, three independent differentiation rounds were performed for each NPC line and three independent images were evaluated in each differentiation round to assess neuronal differentiation efficiency.

### Denaturing SDS-PAGE and WB analysis of neuronal cells

Neuronal cells were homogenized in detergent-free Tris-buffered saline (TBS, 50 mm Tris pH 7.4, 150 mm NaCl) containing 2 mm Ethylenediaminetetraacetic acid (EDTA) und protease inhibitor cocktail (Roche) in a Potter dounce homogenizer on ice. Protein content was determined using a bicinchoninic acid assay (Thermo Fisher Scientific). A total protein of 30 μg of the homogenate were mixed with the equal volume of 2xSDS sample buffer (0.125 M Tris/HCl pH 6.8, 4% SDS, 20% glycerol, 20 mm Dithiothreitol [DTT] and 0.01% bromophenol blue) and separated in a 12% SDS-PAGE gel. In this study, samples were processed for the denaturing SDS-PAGE under reducing conditions (10 mm DTT), which allowed to detect α-Syn and other protein markers in the same samples and in the same WB membranes, when needed. Since α-Syn does not contain any cysteine residues and α-Syn oligomerization is not based on the disulfide bonds between cysteine residues, the use of DTT does not influence the detection of SDS-stable α-Syn species and meanwhile improves the separation and detection of other proteins in WB. For WB, the gel was blotted on a nitrocellulose membrane (Merk Millipore, Darmstadt, Germany) by a semi-dry transfer apparatus (Biometra, Analytik Jena, Jena Germany).

For immunodetection, blotted membranes were fixed for 30 min in 4% PFA to increase the sensitivity of protein detection (58) and washed twice with TBS. To control protein loading, a total protein staining with 0.2% ponceau in 3% acetic acid was performed. The membranes were next blocked with TBS containing 3% bovine serum albumin and probed with primary and horseradish peroxidase-conjugated secondary Abs. For visualization, chemiluminescent substrates (SuperSignal West Chemiluminescent Substrate kits, Thermo Fisher Scientific) were applied to the membranes, and chemiluminescent signals were detected by Gel Doc XR system (Bio-Rad Laboratories, Munich, Germany) and quantified by Image Lab Software (5.2.1, Bio-Rad).

The following primary and secondary Abs were used for WB: monoclonal anti-β-actin (AC-15, #A5441 Sigma-Aldrich, 1:5000), monoclonal mouse anti-Miro1 (CL1083, #ab188029, Abcam, 1:800), polyclonal rabbit anti-nitrotyrosine (#06-284, Upstate, 1:1000), monoclonal mouse anti-α-Syn (Syn1, #610787, BD Biosciences, 1:2000), monoclonal rabbit anti-phosphorylated α-Syn (S129, clone EP1536Y, #ab51253, Abcam, 1:500) and monoclonal mouse anti-β3-tubulin (Tuj1, MMS-435P, Covance, 1:1000).

### Sequential extraction of proteins

α-Syn aggregation was assessed by sequential extraction of proteins, which determined the proportion of insoluble α-Syn, indicative for aggregation. The assay was performed as described previously (12). Briefly, the whole cell homogenate (prepared as described in WB section) was centrifuged at 100 000*g* for 1 h at 4°C and the resulting supernatant was collected as soluble fraction (S1). After re-suspending the pellet in TBS containing 1% Triton X100, followed by a second centrifugation step (100 000*g*, 1 h, 4°C), the Triton X100-soluble fraction (S2) was collected. The Triton X100-insoluble pellet was re-suspended with radioimmunoprecipitation assay (RIPA) buffer (50 mm Tris-HCl, pH 7.4, 175 mm NaCl, 5 mm EDTA, 1% NP-40 and 0.5% sodium deoxycholate containing 1% SDS). RIPA-soluble S3 fraction was collected after a third centrifugation step (100 000*g*, 1 h, 4°C). The remaining RIPA-insoluble pellet (P3) was solubilized in 8 M Urea/5% SDS (P3). α-Syn content within different fractions was analyzed by WB using an anti-α-Syn Ab (Syn1). Intensity of bands corresponding to α-Syn monomers and high molecular weight oligomers were generated by the Image Lab 5.0 software (Biorad).

### ROS measurements

Direct ROS detection was performed in living neurons. To this aim, NPCs from Ctrl and PD Dupl iPSC lines were differentiated to either CPNs or mDANs as described above in 96-well plates with flat-bottom and black walls at a density of 50 × 10^3^ cells per well. CPNs and mDANs were cultured under the same conditions in a minimal essential neuronal media (20 ng/ml brain-derived neurotrophic factor, 20 ng/ml glial cell-derived neurotrophic factor [both from Peprotech], 200 nm ascorbic acid and 1 mm cAMP [both from Sigma-Aldrich]) for the last 48 h to eliminate possible differences in ROS induced by different neuronal differentiation protocols. On the final differentiation day, CPNs and mDANs were stained with 5 μM CellRox Deep Red fluorescence reagent (Thermo Fisher Scientific), which was added directly to the culture medium, for 30 min at 37°C in order to detect cytosolic ROS. To detect the mitochondrial superoxide, cells were pre-washed with warm PBS containing Ca^2+^ and Mg^2+^ (Thermo Fisher Scientific) followed by an incubation with 5 μM MitoSox reagent (Thermo Fisher Scientific) diluted in warm PBS containing Ca^2+^ and Mg^2+^ for 10 min at 37°C. After CellRox and MitoSox treatment, cells were washed once with PBS containing 1 μg/ml DAPI for cell nuclei staining for 5 min at RT and finally washed twice with PBS prior to measurement. CellRox, MitoSox and DAPI fluorescence intensities were measured using a CLARIOstar Plus plate reader (BMG Labtech) at the following Excitation/Emission wavelengths: CellRox - 625-30/680-30 nm; MitoSox - 510-15/580-20 nm; DAPI - 360-20/460-30 nm, respectively. CellRox and MitoSox values in each well were normalized to DAPI to account for cell numbers following by the normalization to the respective values of an external neuronal line, differentiated either with the cortical or with the midbrain protocol, to account for cortical and midbrain cell culture conditions. Each neuronal line was differentiated in quadruplicates for each CellRox and MitoSox detection.

### Statistical methods

Differences among groups were evaluated by one-way analysis of variance (ANOVA) followed by Bonferroni’s or Holm-Sidak multiple comparison tests, while comparisons between two groups were done by two-tailed, paired Student’s *t*-test. P-values ≤ 0.05 were considered significant. Graphs are presented as mean of three independent experiments (*n* = 3) ± standard deviation (SD). Statistical analyses were performed using GraphPad Prism version 5.03 (GraphPad Software, Inc.).

## Abbreviations

Abs, antibodies; ANOVA, analysis of variance; BCA, bicinchoninic acid; BMP, bone morphogenetic protein; C-Casp3, cleaved Caspase 3; CPNs, cortical projection neurons; Ctip2, chicken ovalbumin upstream promoter transcription factor (COUP-TF)-interacting protein 2; Ctrl, control; DA, dopaminergic; DAPI, 4′,6-diamidino-2-phenylindole; DTT, dithiothreitol; Dupl, SNCA duplication; EDTA, ethylenediaminetetraacetic acid; ERK, extracellular-signal-regulated kinase; FGF, fibroblast growth factor; ICC, immunocytochemistry; IF, immunofluorescence; iPSCs, induced pluripotent stem cells; LBs, Lewy bodies; LNs, Lewy neurites; mDANs, midbrain dopaminergic neurons; Miro1, Mitochondrial Rho GTPase 1; MLPA, Multiplex Ligation-Dependent Probe Amplification; NPCs, neural precursor cells; PBS, phosphate-buffered saline; PD, Parkinson’s disease; PFA, paraformaldehyde; RNS, reactive nitrogen species; ROS, reactive oxygen species; RT, room temperature; SD, standard deviation; SDS, sodium dodecyl sulfate; SN, substantia nigra; SNCA, α-synuclein gene; TBS, tris-buffered saline; TGF-β, transforming growth factor β; TH, tyrosine hydroxylase; Tuj1, β3-tubulin; WB, western blot; α-Syn, α-synuclein.
